# Tuning the Exchange Bias Effect in 2D van der Waals Ferro‐/Antiferromagnetic Fe_3_GeTe_2_/CrOCl Heterostructures

**DOI:** 10.1002/advs.202105483

**Published:** 2022-03-03

**Authors:** Tianle Zhang, Yujun Zhang, Mingyuan Huang, Bo Li, Yinghui Sun, Zhe Qu, Xidong Duan, Chengbao Jiang, Shengxue Yang

**Affiliations:** ^1^ School of Materials Science and Engineering Beihang University Beijing 100191 P. R. China; ^2^ Department of Physics Southern University of Science and Technology Shenzhen Guangdong 518055 P. R. China; ^3^ Hunan Key Laboratory of Two‐Dimensional Materials School of Physics and Electronics Hunan University Changsha Hunan 410082 P. R. China; ^4^ Beijing Key Laboratory for Magneto‐Photoelectrical Composite and Interface Science School of Mathematics and Physics University of Science and Technology Beijing Beijing 100083 P. R. China; ^5^ Anhui Key Laboratory of Condensed Matter Physics at Extreme Conditions High Magnetic Field Laboratory Chinese Academy of Sciences Hefei Anhui 230031 P. R. China; ^6^ State Key Laboratory for Chemo/Biosensing and Chemometrics College of Chemistry and Chemical Engineering Hunan University Changsha Hunan 419982 P. R. China

**Keywords:** 2D magnets, exchange bias effect, spintronics, van der Waals heterostructures

## Abstract

The exchange bias effect is extremely expected in 2D van der Waals (vdW) ferromagnetic (FM)/antiferromagnetic (AFM) heterostructures due to the high‐quality interface. CrOCl possesses strong magnetic anisotropy at 2D limit, and is an ideal antiferromagnet for constructing FM/AFM heterostructures to explore the exchange bias effect. Here, the exchange bias effect in Fe_3_GeTe_2_ (FGT)/CrOCl heterostructures through both anomalous Hall effect (AHE) and reflective magnetic circular dichroism (RMCD) measurements is studied. In the AHE measurements, the exchange bias field (*H_EB_*) at 3 K exhibits a distinct increase from ≈150 Oe to ≈450 Oe after air exposure, and such variation is attributed to the formation of an oxidized layer in FGT by analyzing the cross‐sectional microstructure. The *H_EB_* is successfully tuned by changing the FGT/CrOCl thickness and the cooling field. Furthermore, a larger *H_EB_* of ≈750 Oe at 1.7 K in FGT/CrOCl heterostructure through RMCD measurements is observed, and it is proposed that the larger *H_EB_* in RMCD measurements is related to the distribution of uncompensated spins at the interface. This work reveals several intriguing phenomena of the exchange bias effect in 2D vdW magnetic systems, which paves the way for the study of related spintronic devices.

## Graphical abstract

The exchange bias effect in ferro‐/antiferromagnetic Fe_3_GeTe_2_ (FGT)/CrOCl heterostructure is thoroughly studied through anomalous Hall effect and reflective magnetic circular dichroism (RMCD) measurements. The bias field (*H_EB_*) reveals anomaly increase with the presence of an oxidized layer in FGT. The larger *H_EB_* in the RMCD measurements can be related to the distribution of uncompensated spins.

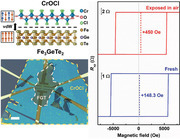

## 1 Introduction

The exchange bias effect is critical for the pinning of ferromagnetic (FM) layer in the magnetic recording and magnetic storage devices. This effect was discovered for the first time in the Co/CoO core‐shell system, and the hysteresis loop of the system was displaced along the magnetic field axis.^[^ ^1^ ^]^ Later, a lot of works have been done on the exchange bias effect in the FM/antiferromagnetic (AFM) thin film, due to its technical significance in spin valves, magnetic read heads, and magnetic random access memories.^[^ ^2^, ^3^, ^4^, ^5^, ^6^ ^]^ The exchange bias is produced by the exchange interaction of the FM/AFM interface, and the quality of the interface plays an important role in determining the exchange bias effect. However, there are still numerous inevitable problems in FM/AFM heterostructures prepared by thin film deposition, such as interdiffusion of atoms and changes in interface composition.^[^ ^7^ ^]^ These phenomena can cause the destruction of the density of states profile in the heterogeneous interface, and further lead to the deterioration of the device performance.^[^ ^8^ ^]^

The discovery of 2D van der Waals (vdW) magnetic materials provides an excellent platform for the study of the exchange bias effect.^[^ ^9^, ^10^ ^]^ Since the intrinsic AFM order in FePS_3_ and the FM order in CrGeTe_3_ were reported,^[^ ^11^, ^12^ ^]^ the family of 2D vdW magnetic materials has been rapidly enriched.^[^ ^13^, ^14^, ^15^, ^16^, ^17^, ^18^, ^19^, ^20^ ^]^ Similar to other 2D vdW materials, the layers in a vdW magnetic material are connected by vdW interaction, which will produce a perfect surface without dangling bonds.^[^ ^21^, ^22^, ^23^ ^]^ Therefore, in the FM/AFM heterostructure assembled from these materials, the interface quality is very high, eliminating surface reconstruction and composition changes. Moreover, the number of 2D vdW magnets increases rapidly, which will expand the choice of material composition in the study of the exchange bias effect.^[^ ^24^ ^]^ In the past 2 years, the exchange bias effect has been discovered in several 2D vdW FM/AFM heterostructures, e.g., Fe_3_GeTe_2_ (FGT)/CrCl_3_ and FGT/MnPS(Se)_3_ heterostructures.^[^ ^25^, ^26^, ^27^ ^]^ Abnormal cooling field dependence of bias field (*H*_*EB*_) and anti‐symmetric magnetoresistance have also been observed in these systems.^[^ ^25^, ^26^ ^]^ Obviously, FGT is widely used as the ferromagnet in the construction of FM/AFM heterostructures because of its extremely high magnetic anisotropy and itinerant ferromagnetism.^[^ ^28^ ^]^ Specially, we here select CrOCl as the AFM component to prepare 2D vdW FM/AFM heterostructures. Compared with the antiferromagnets used in previous works, CrOCl belongs to a totally different material family, i.e., transition metal oxyhalides. The bulk materials in this family show plentiful phase transition behavior, e.g., magnetoelastic coupling and spin‐Peierls transition,^[^ ^29^, ^30^ ^]^ illustrating that there will be novel physical mechanism in these materials at 2D limit. As a representative material of this family, few‐layer CrOCl possesses strong magnetic anisotropy, making it suitable for research on the exchange bias effect.^[^ ^31^ ^]^ Moreover, CrOCl has unique optical anisotropy, allowing it to expand to a wider range of applications, such as optical spintronics.^[^ ^32^ ^]^ Furthermore, in previous reports, the exchange bias effect was characterized by either electrical methods (anomalous Hall effect (AHE) measurement) or optical methods (magneto‐optical Kerr effect/magnetic circular dichroism (MCD) measurement), so it is necessary to investigate the impact of detection methods on the characterization of the exchange bias effect.

In this work, we fabricate FGT/CrOCl heterostructures by polymer‐based dry transfer technique, and characterize the exchange bias effect in this system using AHE and reflective magnetic circular dichroism (RMCD) measurements. In the AHE measurements, the exchange bias effect disappears near the Neel temperature (*T_N_*) of CrOCl, and the direction of the *H_EB_* is always opposite to the direction of the cooling field, indicating that there is a negative exchange bias effect in this system. Besides, the *H_EB_* reaches ≈150 Oe at 3 K, and further increases to ≈450 Oe after exposing the fresh device to ambient conditions for several days, due to the formation of an oxidized layer in the FGT through cross‐sectional microstructure analysis. By changing the thickness of the FGT/CrOCl and the cooling field, the magnitude of *H_EB_* is successfully modulated, which is the result of the change in the strength of the interface exchange interaction. In the RMCD measurements, the exchange bias effect disappears at a temperature higher than the *T_N_* of CrOCl, and a considerable *H_EB_* of 750 Oe is observed at 1.7 K in the FGT/CrOCl heterostructure, where the larger *H_EB_* is related to the distribution of uncompensated spins at the interface.

**Figure 1:**
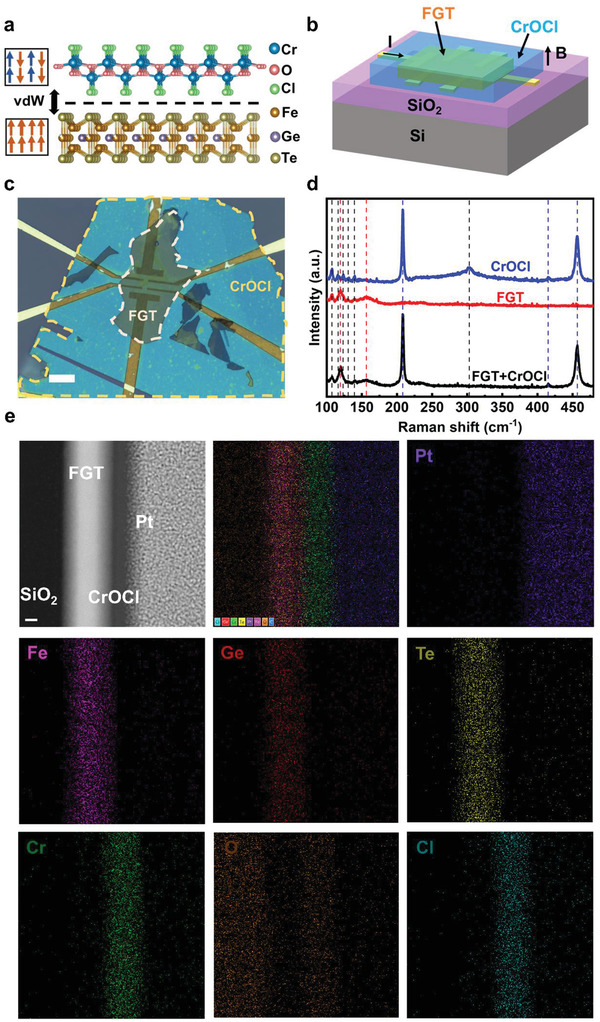
a) Crystal structure and magnetic order of FGT and CrOCl. b) Schematic diagram of the device structure of the FGT/CrOCl heterostructure. c) OM image of device 1. Scale bar is 10 µm. d) Raman spectra of individual FGT, individual CrOCl, and stacked FGT/CrOCl heterostructure. The Raman peaks of FGT/CrOCl/substrate are denoted by red/blue/black dashed lines, respectively. e) Cross‐sectional scanning transmission electron microscope (STEM) image and corresponding element mapping of fresh FGT/CrOCl heterostructure. Scale bar is 10 nm.

The synthesis of FGT and CrOCl single crystals has been elaborated in our previous works.^[^ ^32^, ^33^ ^]^ FGT is a typical ferromagnet with strong out‐of‐plane magnetic anisotropy. This material belongs to *P6_3_/mmc* space group, and each layer consists of five atomic sublayers, where a Fe_3_Ge slab is sandwiched between two Te atom layers (bottom part of **Figure** **1**a). CrOCl exhibits antiferromagnetism at low temperatures with an out‐of‐plane easy axis. It belongs to *Pmmn* space group, and the basal plane is made up of corrugated CrO layer sandwiched by two Cl atom layers (top part of Figure 1a).

To minimize the contamination of our samples caused by chemical residues in the fabrication of electrodes, we adopt the device structure shown in Figure 1b. First, we prepatterned the Ti/Au (5/30 nm) electrodes on a SiO_2_ (285 nm)/Si substrate. In a glove box filled with Ar atmosphere, a FGT flake was then transferred onto the electrodes using polymer‐based dry transfer technique,^[^ ^25^ ^]^ and a CrOCl flake was subsequently transferred onto the FGT flake to form a FM/AFM heterostructure. The insulation of CrOCl ensures that FGT is used as the dominant conducting channel in the AHE measurements. Figure 1c displays the optical microscope (OM) image of a representative device (device 1) used for the AHE measurements, in which the thickness of FGT/CrOCl is ≈27/28 nm (Figure S1, Supporting Information), respectively.

To further verify the quality of the heterostructure, we measured Raman spectra on the regions of stacked heterostructure, as well as individual FGT and CrOCl flakes. As shown in Figure 1d, after subtracting the Raman peaks of the substrate (Figure S2, Supporting Information), the Raman peaks of the stacked heterostructure are the sum of the Raman peaks of the individual FGT and CrOCl flakes, with no additional peaks. Moreover, we conducted elemental analysis on the cross‐sectional sample of the heterostructure using energy‐dispersive X‐ray spectroscopy (EDS). From the line scan and element mapping results (Figure S3, Supporting Information; Figure 1e), the Fe, Ge, Te and Cr, O, Cl elements are uniformly distributed in the corresponding region, and a clear interface is noticed between FGT and CrOCl layers.

**Figure 2:**
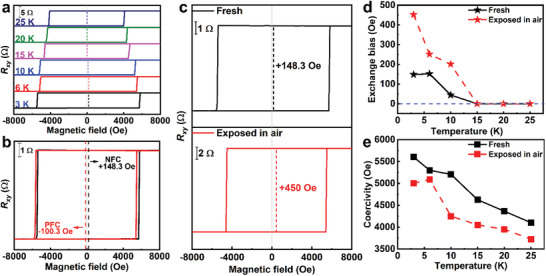
a) Field‐dependent *R_xy_* of device 1 measured under the temperature range from 3 to 25 K. b) Field‐dependent *R_xy_* measured at 3 K under PFC/NFC. c) Field‐dependent *R_xy_* of fresh/air‐exposed (4 days) devices measured at 3 K under NFC. Temperature dependence of *H_EB_* d) /*H_C_* e) of fresh and air‐exposed (4 days) devices.

**Figure** **2** presents the AHE measurement results of device 1 (Figure 1c). The field‐dependent Hall resistance *R_xy_* of the fresh device was measured at different temperatures. The cooling field was kept at −1 T, and the direction of cooling field was perpendicular to *a‐b* plane of device. As depicted in Figure 2a, the exchange bias effect disappears when the temperature exceeds 15 K, which is consistent with the *T_N_* of CrOCl (13.7 K).^[^ ^34^ ^]^ In the whole temperature range, the direction of *H_EB_* is positive, opposite to the cooling field (−1 T), that is, the negative exchange bias effect exists in this system, which confirms the FM coupling at the interface.^[^ ^35^, ^36^ ^]^ This is further verified in Figure 2b, where bias fields of +148.3 Oe and −100.3 Oe are observed at 3 K under −1 T negative field cooling (NFC) and +1 T positive field cooling (PFC). The *H_EB_* is relatively small, but still comparable to the *H_EB_* obtained in the FGT/MnPS(Se)_3_ heterostructure.^[^ ^26^ ^]^ Specially, the magnitude of *H_EB_* presents a great increase from ≈150 Oe to ≈450 Oe (Figure 2c) after air exposure (4 days), while the direction of *H_EB_* remains opposite to the direction of the cooling field (Figure S4, Supporting Information). With the increase of exposure time from 2 to 4 days, the *H_EB_* increases while the coercivity (*H_C_*) decreases monotonically (Figure S5, Supporting Information). As for the temperature‐dependent measurements, the *H_E__B_* and *H_C_* are extracted from the hysteresis loops measured on the fresh and air‐exposed samples to establish a direct comparison between these properties. As shown in Figure 2d,e, the *H_EB_*/*H_C_* of the device after air exposure is larger/smaller than that of the fresh device in the whole temperature range, respectively. Additionally, the *H_EB_* of the air‐exposed device also disappears at 15 K, indicating that such an exchange bias effect also arises from the interface exchange interaction between FGT and CrOCl.

**Figure 3:**
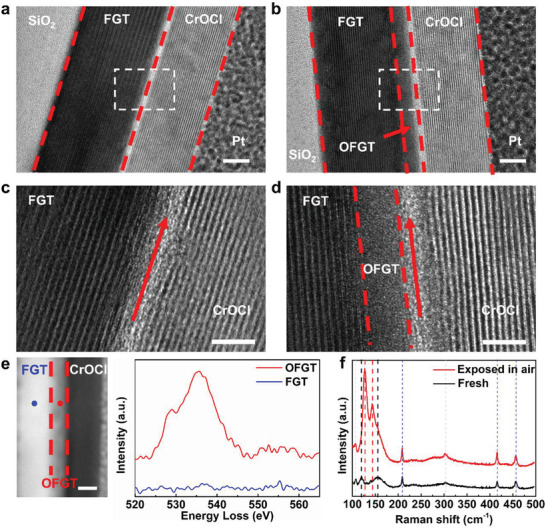
Low‐magnification cross‐sectional HRTEM image of a) the fresh device and b) the device exposed in air for 4 days. High‐magnification cross‐sectional HRTEM image of the part marked by white dashed rectangle in (a,c) and in (b,d). The interface between FGT and CrOCl is indicated by the red arrow. e) Cross‐sectional STEM image (left) and corresponding O‐K spectra (right) of the device exposed in air for 4 days. The red/blue lines are the measurement results of the red/blue dots in the cross‐sectional STEM image, respectively. f) Raman spectra of fresh/air‐exposed FGT/CrOCl heterostructure. Raman peaks of FGT in fresh/air‐exposed sample are denoted by black/red dashed lines, respectively. Raman peaks of CrOCl/substrate are denoted by blue/gray dashed lines, respectively. All scale bar is 10 nm.

To elucidate the origin of the change of *H_EB_* and *H_C_* after air exposure, we acquired the cross‐sectional samples of the fresh and air‐exposed (4 days) devices by focus ion beam (FIB), and then analyzed the microstructure of these samples via high‐resolution transmission electron microscope (HRTEM). **Figure** **3**a,b are the low‐magnification cross‐sectional HRTEM images of the fresh and air‐exposed devices, respectively. In the fresh one, clear layered structure is observed through the whole region of FGT and CrOCl. In comparison, it is noticeable that in FGT, an amorphous layer appears near the FGT/CrOCl interface in the air‐exposed device (as denoted by the red arrow in Figure 3b), and the structure difference of these two samples is more evident in the high‐magnification HRTEM images (Figure 3c,d). Combined with the elemental analysis on the cross‐sectional sample of the air‐exposed device, such amorphous layer is identified as the oxidized FGT. Figure 3e illustrates the O‐K edge spectra of the amorphous layer and the inner FGT acquired by electron energy loss spectroscopy (EELS). The obvious peak in the O‐K edge spectra of the amorphous layer provides direct evidence for the formation of oxidized FGT layer, and the vanishing O intensity in the inner FGT region confirms that oxidation only occurs at the surface of FGT. Besides, elemental analysis is also conducted on the cross‐sectional sample using EDS. In the element mapping images of the fresh sample, the O content in the FGT region is very limited, which is determined by the resolution of EDS (Figure 1e; Figure S3, Supporting Information). However, in the elemental analysis of the air‐exposed sample (Figures S6 and S7, Supporting Information), considerable O distributes in the FGT region near the FGT/CrOCl interface. For the sample exposed to air for a longer time (1 week; Figures S8 and S9, Supporting Information), the amorphous layer is much thicker, and the O distribution region in the FGT is deeper as well, which further verifies that the amorphous layer is oxidized FGT.

Moreover, the presence of oxidized FGT can also be detected by Raman spectra. As shown in Figure 3f, the Raman peaks of FGT are located at 119.6 cm^−1^ and 155.5 cm^−1^ in the fresh sample. After air exposure, these two peaks shift to 127.4 cm^−1^ and 143.9 cm^−1^, respectively, which confirms the degradation of FGT.^[^ ^37^ ^]^ Furthermore, if the device is exposed to air for even longer, the formation of oxidized layer can be directly observed through the OM image. Figure S10 (Supporting Information) displays the comparison of the OM images of a device before and after being exposed to the air for 1 month. Apparently, the thin area of the FGT flake is almost invisible after air exposure, which is caused by the formation of oxidized FGT layer.

Based on the well‐known Meiklejohn–Bean model,^[^ ^1^ ^]^ the *H_EB_* for this system can be written as Equation (1):

(1) $$H_{EB}=\;-\frac{J_{AFM-FM}}{\mu_{0}*t_{FM}*M_{FM}}$$

where *J_AFM‐FM_* is the strength of the interface exchange coupling between ferromagnet and antiferromagnet, *t_FM_* is the thickness of FM layer, *M_FM_* is the saturated magnetization of FM layer, and *μ*_0_ is the vacuum permeability. For the air‐exposed device, *t_FM_* and *M_FM_* should not change significantly because the oxidized layer is relatively thin compared with the whole FGT. As a result, the variation of *H_EB_* is mainly related to *J_AFM‐FM_*, that is, the presence of oxidized FGT layer increases the interface coupling strength between FGT and CrOCl, and further increases the *H_EB_*. As previous reports show, the interfacial exchange interaction depends strongly on the spin configuration at the interface, which is further related to several intrinsic and extrinsic factors, such as the anisotropy, uncompensated spins at the antiferromagnet interface, and the interface roughness.^[^ ^2^ ^]^ A previous work has shown that the interfacial magnetic anisotropy will increase with the formation of oxidized FGT, which will contribute to the increase of interfacial interaction.^[^ ^38^ ^]^ Moreover, it is shown that the surface roughness of FGT increases a little with the formation of oxidized layer (Figure S11, Supporting Information), and this may also enhance *J_AFM‐FM_* through increasing the uncompensated spins at the interface.^[^ ^39^ ^]^ In recent reports, topological magnetic textures such as skyrmions have been observed in the FGT with an oxidized layer,^[^ ^40^ ^]^ which reveals the intriguing properties of oxidized FGT. In addition, AHE measurements have been conducted on naturally oxidized FGT in a previous work,^[^ ^41^ ^]^ and no exchange bias effect is noticed, which further validates that the effect of FGT/oxidized FGT layer itself contributes little to the exchange bias effect in the air‐exposed device here.

The magnitude of *J_AFM‐FM_* can also be estimated using Equation (1), from which it is proportional to *t_FM_*, *H_EB_*, and *M_FM_*. The former two parameters can be determined in our experiments, while it is difficult to obtain the accurate value of *M_FM_*. Considering that the FGT used in our experiments are relatively thick (most > 20 nm), it is reasonable to use the *M_FM_* of bulk FGT as an estimation, which is around 376 emu cm^−3^.^[^ ^42^ ^]^ Consequently, it is derived that the magnitude of *J_AFM‐FM_* increases from around 0.14 to 0.46 mJ m^−2^ with the formation of oxidized layer, while the latter one is comparable to the interface interaction in the Co/CoO bilayer system.^[^ ^36^ ^]^ Moreover, the estimated *J_AFM‐FM_* of all the samples used in our work has been summarized in the Table S1 (Supporting Information).

**Figure 4:**
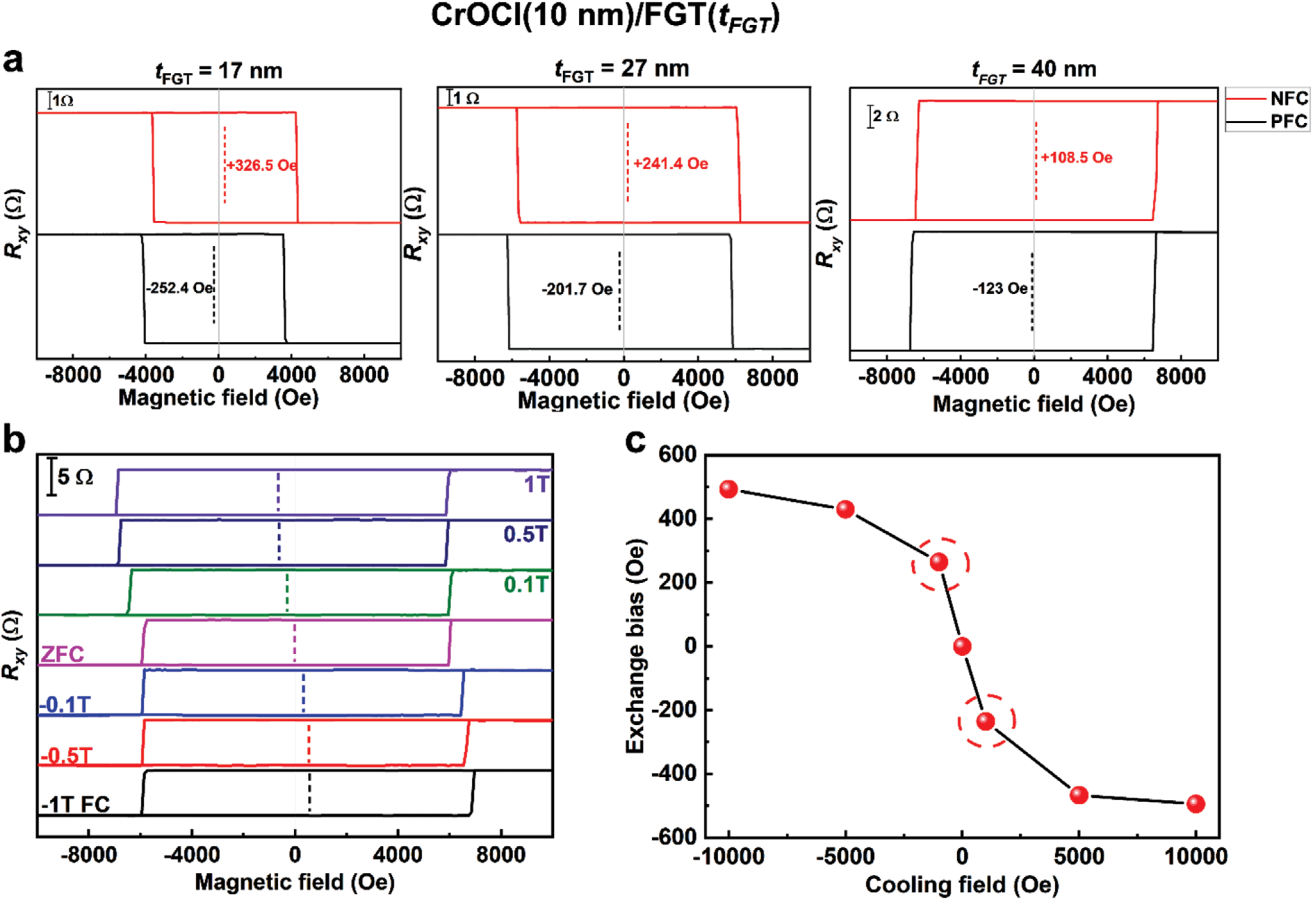
a) Field‐dependent *R_xy_* measured at 3 K on devices with CrOCl of identical thickness (10 nm) and FGT of different thicknesses (17 nm in the left panel of (a), 27 nm in the middle panel of (a), and 40 nm in the right panel of (a)). Both NFC and PFC are adopted. b) Field‐dependent *R_xy_* of device two after air exposure measured at 3 K under different cooling fields. c) *H_EB_* at 3 K extracted from b) as a function of cooling field. The *H_EB_* under the cooling field of ±0.1 T is marked by the red dashed circle.

From Equation (1), the thickness of FGT may also influence the *H_EB_*. As shown in **Figure** **4**a, the thickness of CrOCl is fixed at around 10 nm. As the thickness of FGT increases from 17 to 27 nm then to 40 nm, the *H_EB_* changes from +326.5 Oe to +241.4 Oe then to +108.5 Oe under NFC, and it changes from −252.4 Oe to −201.7 Oe then to −123 Oe under PFC. The magnitude of *H_EB_* slightly increases with the decrease of FGT thickness, and the thickness dependence of *H_EB_* fits well with Equation (1) (Figure S12, Supporting Information). Considering that the exchange bias effect is an interface effect, with the increase of *t_FM_*, the effect of the interface exchange interaction on the ferromagnet will be weakened, which leads to the decrease of the *H_EB_*. Besides, since the thickness of antiferromagnet (*t_AFM_*) is also a crucial parameter for *J_AFM‐FM_*, *t_AFM_* may affect the magnitude of *H_EB_* as well.^[^ ^43^ ^]^ When the FGT thickness is fixed at 27 nm, the *H_EB_* slightly increases from +148.3 Oe (−100.3 Oe) to +241.4 Oe (−201.7 Oe) under NFC (PFC) with the decrease of *t_AFM_* from 28 nm (Figure 2b) to 10 nm (Figure 4a), and similar results have also been observed in the FGT/CrCl_3_ heterostructure.^[^ ^25^ ^]^

The cooling field is another important factor in determining the *H_EB_*. To investigate the effect of the cooling field, we first measured the initial magnetization curve of another FGT/CrOCl heterostructure device (device 2), in which the thickness of FGT/CrOCl was ≈35/12 nm, respectively. Figure S13(Supporting Information) shows a typical measurement result conducted at 3 K under zero field cooling (ZFC). From the initial magnetization curve, *R_xy_* remains almost unchanged when the magnetic field changes from zero to around −1 kOe, then it changes abruptly with magnetic field and saturates at around −2 kOe. Hereafter, we measured the *H_EB_* of device 2 after air exposure at 3 K under different cooling fields. As illustrated in Figure 4b, negative exchange bias effect arises in the whole measurement, and the magnitude of *H_EB_* increases with the change of cooling field from 0.1 (−0.1 T) to 1 T (−1 T), while no exchange bias effect is observed under ZFC. Moreover, it is clear that the decrease of *H_EB_* is larger when the cooling field is reduced from 0.5 to 0.1 T (>200 Oe) than from 1 to 0.5 T (<100 Oe), as shown in Figure 4c. It has been reported that the cooling field will mainly affect the magnetization state of ferromagnet in the cooling process, further altering the *J_AFM‐FM_* and the *H_EB_* subsequently.^[^ ^44^ ^]^ As for the system discussed here, when the cooling field is reduced from 1 to 0.5 T, the magnetization of FGT stays near saturation, and the *H_EB_* will show little decrease with the change of cooling field. In comparison, when reducing the cooling field from 0.5 to 0.1 T, the magnetization of FGT exhibits a noticeable reduction according to the initial magnetization measurements, which further causes the distinct decrease of *H_EB_*. At the end of the AHE measurements, we measured the *R_xy_* of a pure FGT (35 nm) (Figure S14, Supporting Information). It can be seen that there is no exchange bias effect for pure FGT, further validating the indispensable role of CrOCl.

**Figure 5:**
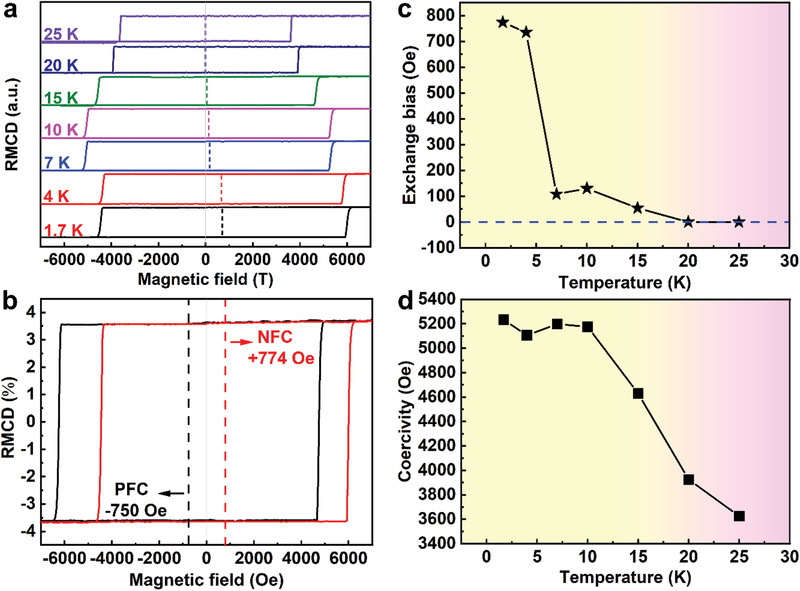
a) Field‐dependent RMCD signals measured at different temperatures under a cooling field of −1 T. b) Field‐dependent RMCD signals measured at 3 K under PFC/NFC. Temperature dependence of c) *H_EB_* and d) *H_C_* extracted from (a).

RMCD was also used to investigate the exchange bias effect in the FGT/CrOCl heterostructure in the form of more microscopic regions. **Figure** **5**a shows the hysteresis loops of a typical FGT/CrOCl heterostructure (point A in Figure S15, Supporting Information) measured in the temperature range of 1.7–25 K, and it can be seen that negative exchange bias effect is also observed in the RMCD measurements. However, the *H_EB_* in the current test area reaches +774 Oe/−750 Oe at 1.7 K under NFC/PFC, respectively, which is much higher than the *H_EB_* in the AHE measurements (Figure 5b). With the increase of temperature, both the *H_EB_* and the *H_C_* decrease monotonically (Figure 5c,d). Nonetheless, a small *H_EB_* is still observed at 15 K, while it disappears at 20 K, and this is also distinct from the AHE measurements in which the *H_EB_* is already zero at 15 K. The AHE measurements will read out the magnetic information of the whole device, while the RMCD measurements focus on a rather small region of the sample, as a result, the magnetic microstructures will have different impacts when using these two detection methods.^[^ ^45^ ^]^ There are two differences between the RMCD and AHE measurement results, the first one is the larger *H_EB_* of certain areas in the RMCD measurements (Figure S16, Supporting Information), and the second one is that the exchange bias effect disappears at a higher temperature in the RMCD measurements. We assume that the former phenomenon is related to the distribution of uncompensated spins at the AFM interface, and the small detection scope of RMCD measurements enables the detection of exchange bias effects in regions with concentrated distribution of uncompensated spins.^[^ ^2^, ^46^, ^47^ ^]^ As for the latter phenomenon, previous work has shown that short‐range magnetic order may exist in CrOCl in the temperature range of ≈14–28 K, which means that a weak magnetic interaction may also exist at the FM/AFM interface in this temperature range.^[^ ^34^ ^]^ In the RMCD measurements, such weak interface interaction becomes detectable because of the rather small detection region. Furthermore, we performed the RMCD measurements on a CrOCl/FGT/CrOCl region (point B in Figure S15, Supporting Information). As shown in Figure S17 (Supporting Information), we can see a larger *H_EB_* of around 900 Oe at 1.7 K and a more obvious exchange bias effect at 15 K, demonstrating that the interface exchange interaction from both sides of the FGT will cause a stronger exchange bias effect. Likewise, the RMCD measurements were also conducted on a pure FGT covered by *h*‐BN at different temperatures, and no exchange bias effect is observed as well (Figure S18, Supporting Information).

## 2 Conclusion

In conclusion, we have combined AHE with RMCD measurements to reveal the exchange bias effect in the FGT/CrOCl heterostructure, especially comparing the *H_EB_* of the fresh device with that of the air‐exposed device. In the AHE measurements, the *H_EB_* increases from ≈150  to ≈450 Oe at 3 K with the formation of a thin oxidized layer in FGT, probably due to the enhancement of interface magnetic interaction, which illustrates the presence of distinctive physics in oxidized FGT. In addition, the exchange bias effect can be modulated well by changing the FM/AFM thickness and the cooling field. In the RMCD measurements, a larger *H_EB_* of around 750 Oe is obtained in the FGT/CrOCl heterostructure at 1.7 K, and the exchange bias effect vanishes at a temperature higher than the *T_N_* of CrOCl, which is relevant to the distribution of uncompensated spins and the smaller detection scope of RMCD measurements. This work clearly demonstrates several unique properties of the exchange bias effect in the 2D vdW FM/AFM heterostructure, which is significant for the application of 2D vdW magnets in the spintronic devices, and our work will also trigger the experimental and theoretical interests toward transition metal oxyhalides family to unveil the intriguing properties of these materials at 2D limit. Additionally, the fabrication of large‐area 2D vdW FM/AFM heterostructures remains a challenge in the industrial processing, and the development of the growth of large‐area 2D vdW magnets together with the large‐area high‐quality transfer method will facilitate the application of 2D vdW FM/AFM heterostructures in the spintronics.

## 3 Experimental Section

### Device Fabrication

First, a six‐electrode Hall bar was prepatterned on the SiO_2_ (285 nm)/Si substrate through using electron beam lithography (EBL) followed by the deposition of Ti/Au (5/30 nm) electrodes. Then, FGT and CrOCl were exfoliated onto the polydimethylsiloxane (PDMS) stamps, and sequentially transferred onto the electrodes to fabricate the device. The exfoliation and transfer processes were conducted in a glove box filled with an Ar atmosphere (O_2_ < 0.1 ppm, H_2_O < 0.1 ppm).

### Cross‐Sectional Microstructure Analysis

The cross‐sectional samples were first obtained from the fresh and air‐exposed FGT/CrOCl heterostructures using FIB (Zeiss crossbeam 540). The microstructure analysis was then carried out on the cross‐sectional samples using HRTEM (Tecnai G2 F20 S‐Twin), in which the accelerating voltage was 200 kV. The element analysis was conducted using the EDS equipped on the HRTEM. The EELS spectra were acquired using a Gatan energy filter (Quantum 963) equipped on a TEM (FEI Titan G2 80–300 ETEM microscope) under an acceleration voltage of 300 kV.

### Magneto‐transport Measurements

The AHE measurements were conducted in the Physical Property Measurement System (DynaCool, Quantum Design). The temperature was first raised above the *T_N_* of CrOCl, and then the sample was cooled under a constant magnetic field. In the measurements, the magnetic field was perpendicular to the sample plane, and a constant current of 100 µA was applied. Compared to the *R_xy_*, the longitudinal magnetoresistance can be ignored. Therefore, the data processing methods used in previous work were adopted.^[^ ^26^ ^]^

### RMCD Measurements

RMCD was performed in an autoDRY 2100 cryostat with the temperature ranges from room‐temperature to 1.7 K. Through using free‐space optics, a HeNe laser was coupled to the cryostat, of which the wavelength was 633 nm. First, the laser beam was tuned using a chopper with a frequency of 800 Hz. Then, it passed through a photoelastic modulator (PEM), and the frequency was at 50.2 kHz with a maximum retardance of *λ*/4. Through using a low‐temperature objective, the modulated laser beam was focused onto the sample, and the power was around 2 µW. Finally, the reflected light was collected using the same objective and it was further detected by a photodiode. The MCD signal was determined as a ratio of the ac component at 50.2 kHz and the dc component (both of them were measured by lock‐in amplifiers) of the reflected light intensity.

### Raman Measurements

The Raman spectra were collected in a backscattering configuration using a LabRAM HR Evolution spectrometer (Horiba Jobin‐Yvon), and the spectrometer was equipped with a liquid‐nitrogen‐cooled charge‐coupled device (CCD) and volume Bragg gratings. The wavelength of the incident laser was kept at 532 nm, and the spot size was kept at around 1 × 1 µm^2^. The scattered light was collected using an objective with 50× focus‐length, and it was dispersed with an 1800 grooves mm^−1^ grating. The laser power was kept at ≈150 µW, and the focus time was kept at ≈90 s.

### Statistical Analysis

The intensities of Raman data in Figure 1d, Figure 3f, and Figure S2 (Supporting Information), the intensities of EELS data in Figure 3e, and the intensities of RMCD data in Figure 5a and Figure S16–S18 (Supporting Information) were normalized. The statistical analysis was carried out using Microsoft Office Excel and Origin software.

## Conflict of Interest

The authors declare no conflict of interest.

## Supporting information

Supporting Information

## Data Availability

The data that support the findings of this study are available from the corresponding author upon reasonable request.
