# Supplementary material for: Tuning the Exchange Bias Effect in 2D van der Waals Ferro‐/Antiferromagnetic Fe3GeTe2/CrOCl Heterostructures
Source: Adv Sci (Weinh). 2022 Mar 3;9(11):2105483. doi: 10.1002/advs.202105483 (PMC9009105; doi:10.1002/advs.202105483)
Supplement: Supplementary file 1 — Supporting Information [file ADVS-9-2105483-s001.pdf]

ADVANCED SCIENCE
Open Access

Supporting Information

for *Adv. Sci.*, DOI 10.1002/advs.202105483

# Tuning the Exchange Bias Effect in 2D van der Waals Ferro-/Antiferromagnetic $Fe_3GeTe_2$/CrOCl Heterostructures

*Tianle Zhang, Yujun Zhang, Mingyuan Huang, Bo Li, Yinghui Sun, Zhe Qu, Xidong Duan, Chengbao Jiang and Shengxue Yang**

# Supporting Information

## Tuning the Exchange Bias Effect in 2D van der Waals Ferro-/Antiferromagnetic $Fe_3GeTe_2$/CrOCl Heterostructures

*Tianle Zhang, Yujun Zhang, Mingyuan Huang, Bo Li, Yinghui Sun, Zhe Qu, Xidong Duan, Chengbao Jiang, Shengxue Yang**

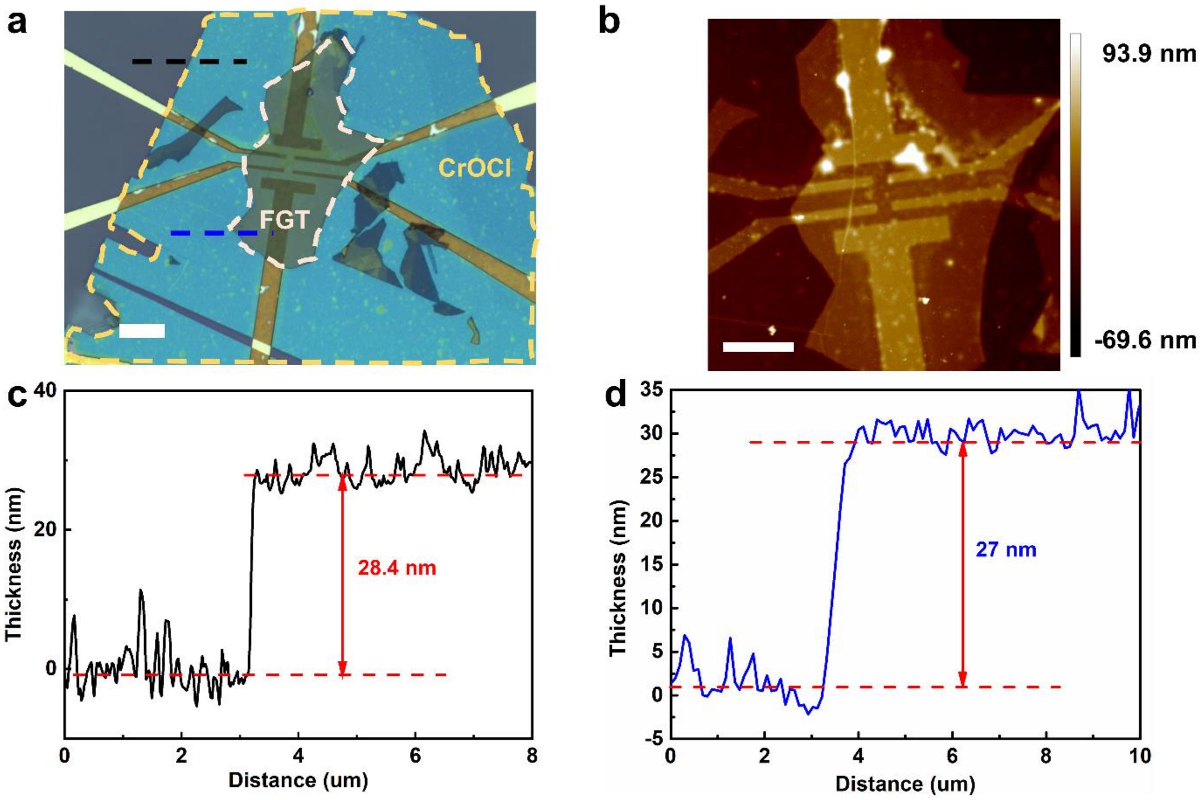


**Figure S1.** a) OM image of the representative device used for the AHE measurements. b) Atomic force microscope image of the device shown in Figure S1a. c) Cross-sectional scanning along the black dashed line in Figure S1a. d) Cross-sectional scanning along the blue dashed line in Figure S1a. All scale bar is 10 μm.

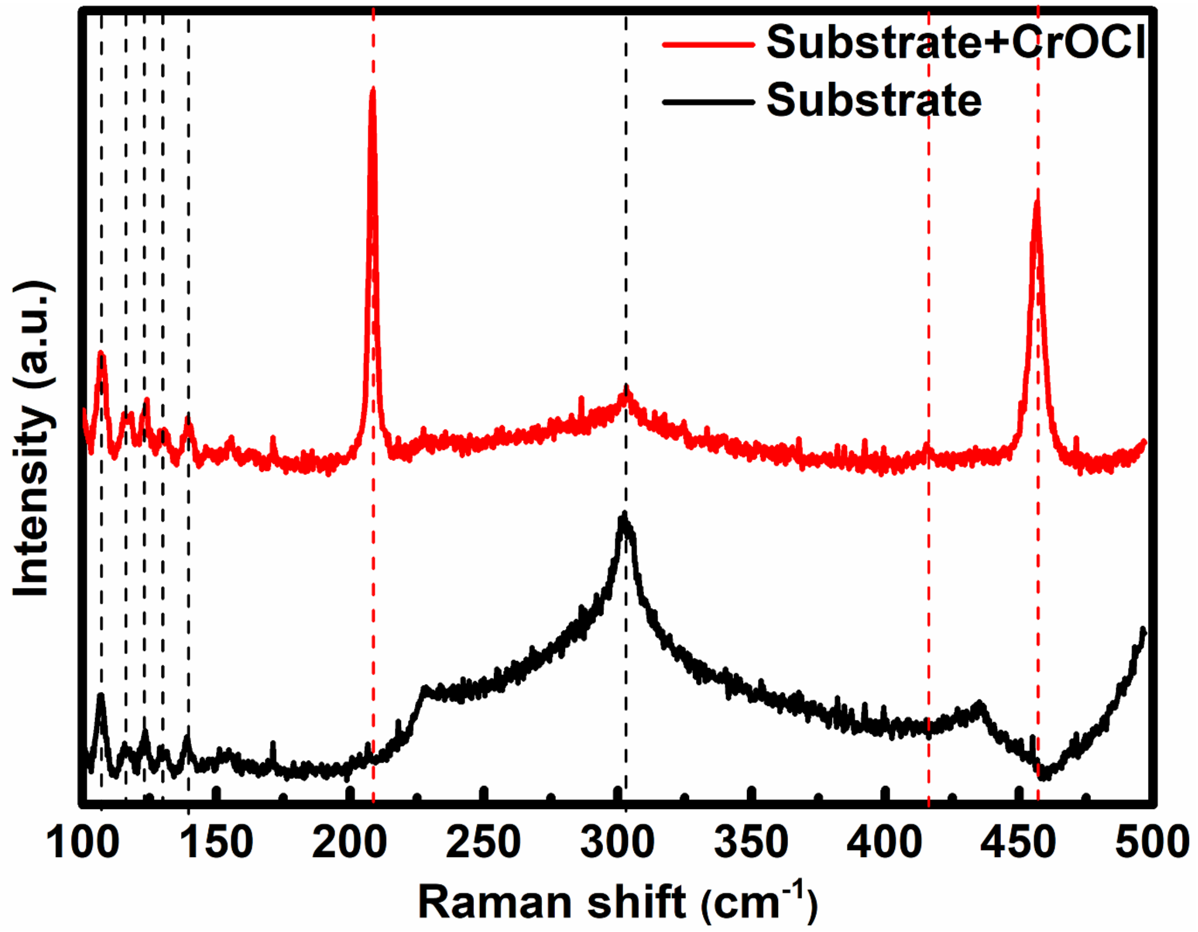


**Figure S2.** Raman spectra of substrate and substrate + CrOCl. Raman peaks of substrate and CrOCl are denoted by black dashed lines and red dashed lines, respectively.

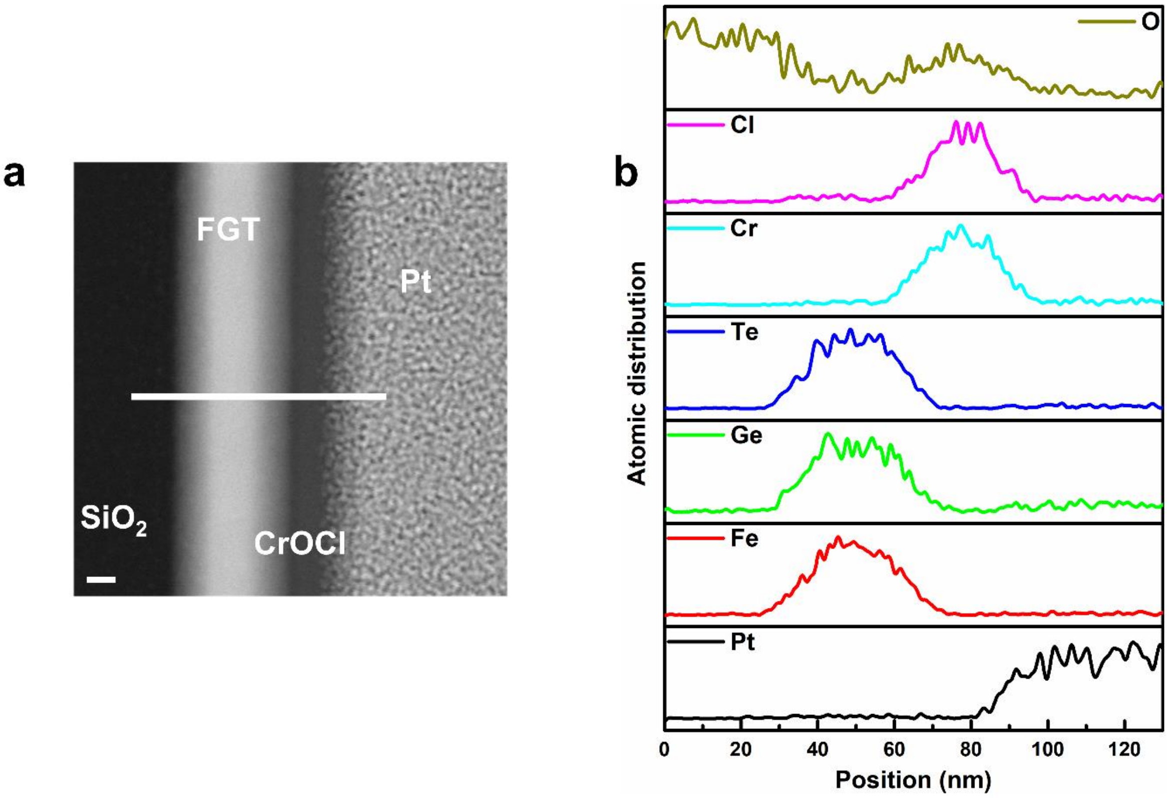


**Figure S3.** a) Cross-sectional STEM image of cross-sectional sample of fresh FGT/CrOCl heterostructure. b) Element distribution along the white line in Figure S3a. Scale bar is 10 nm.

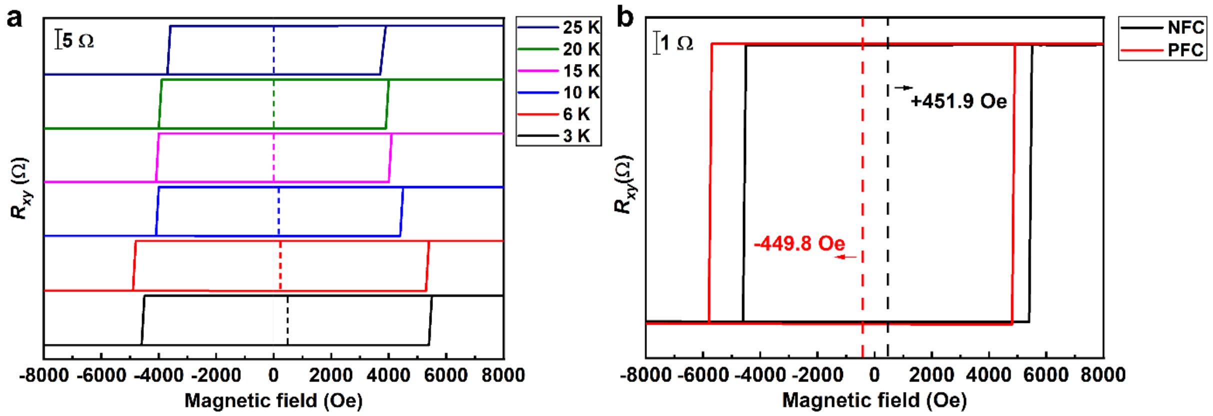


**Figure S4.** a) Hysteresis loops of the device exposed in air for 4 days at different temperatures under the cooling field of -1 T. b) Hysteresis loops of the device exposed in air for 4 days at 3 K under NFC/PFC.

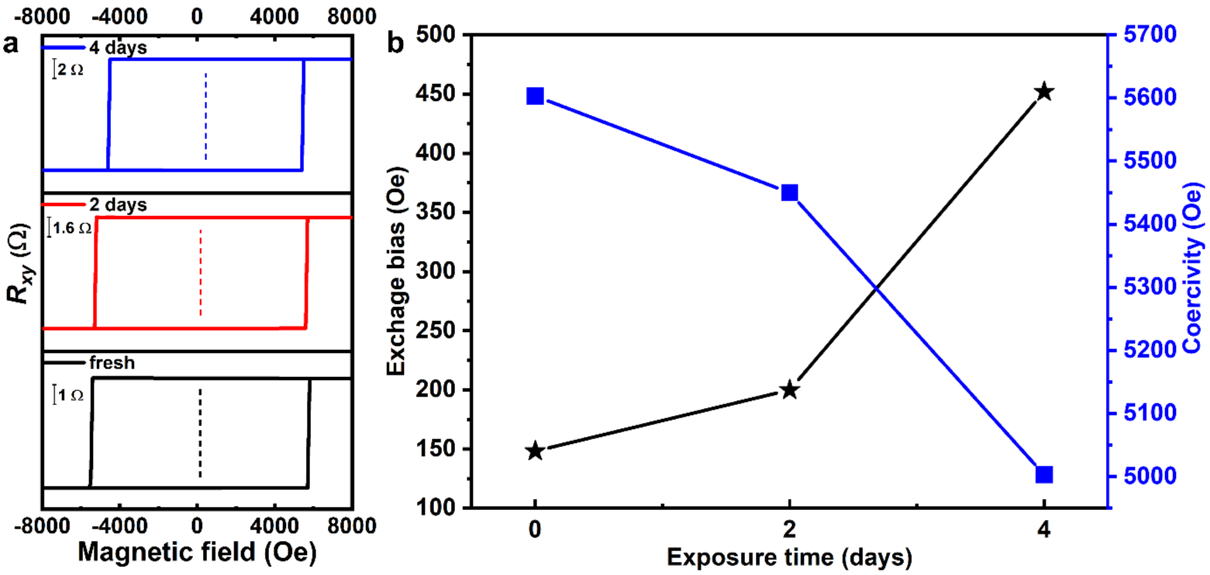


**Figure S5.** a) Hysteresis loops of the fresh device, and the devices exposed in air for 2 days and 4 days at 3 K under the cooling field of -1 T. b) Coercivity and exchange bias field as a function of exposure time in air.

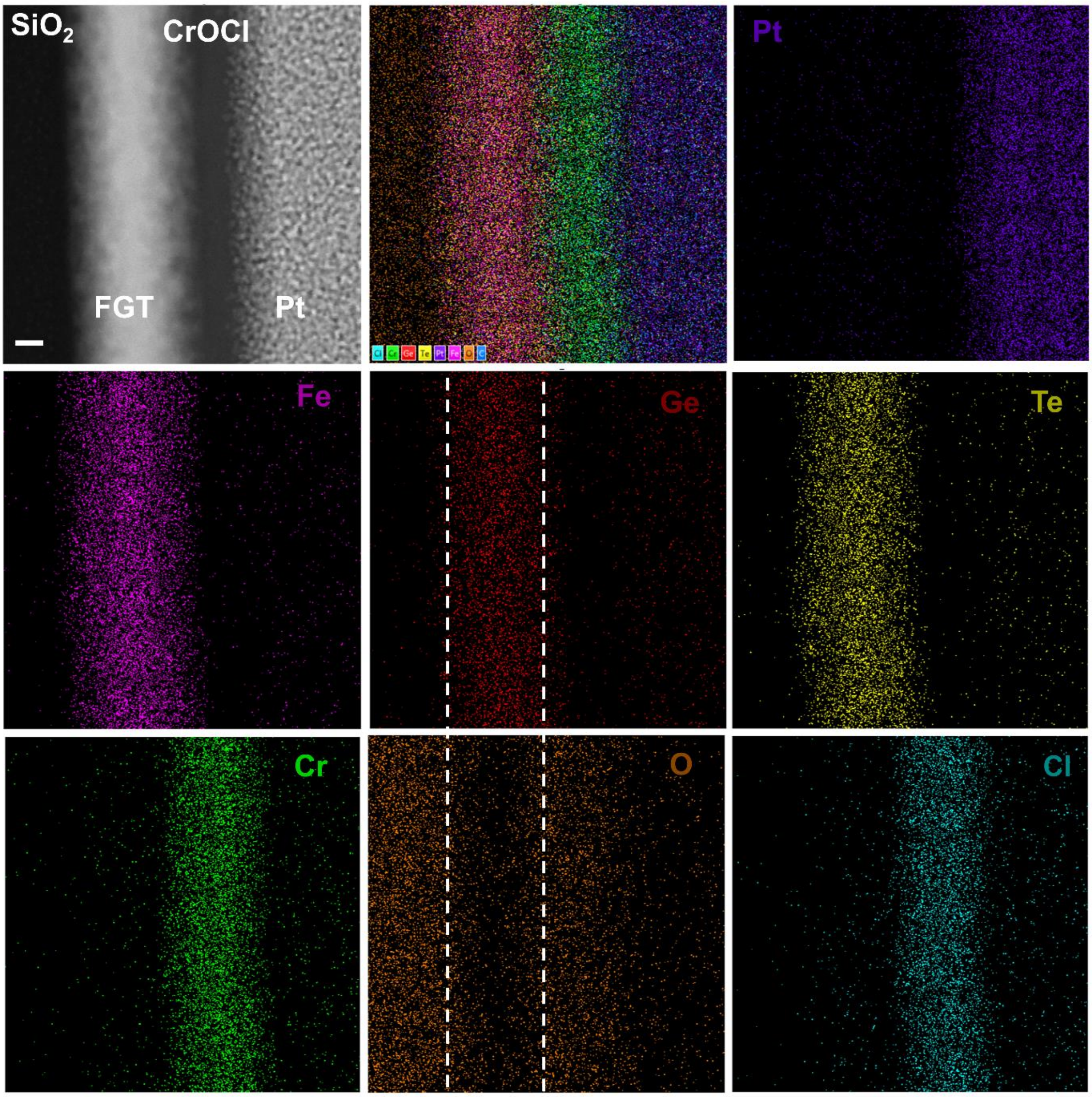


**Figure S6.** Cross-sectional STEM image and corresponding element mapping of FGT/CrOCl heterostructure exposed in air for 4 days. Scale bar is 10 nm.

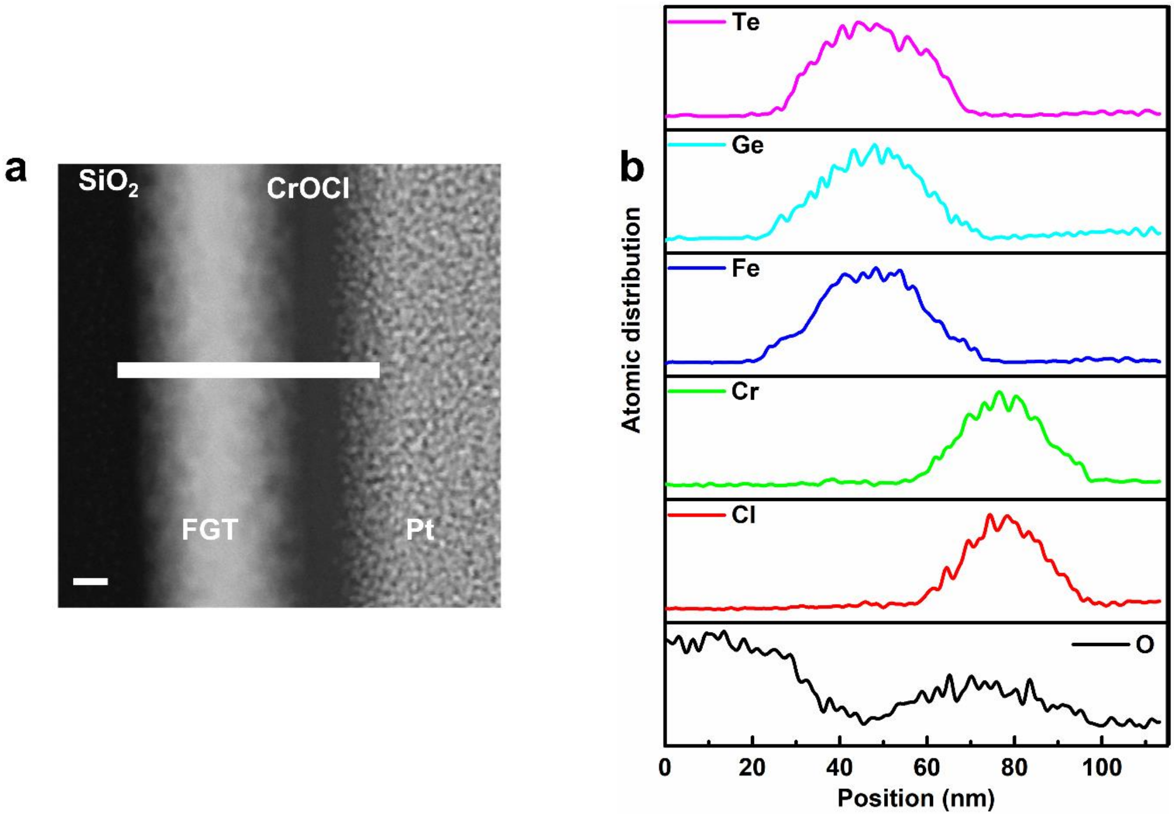


**Figure S7.** a) Cross-sectional STEM image of FGT/CrOCl heterostructure exposed in air for 4 days. b) Element distribution along the white line in Figure S7a. Scale bar is 10 nm.

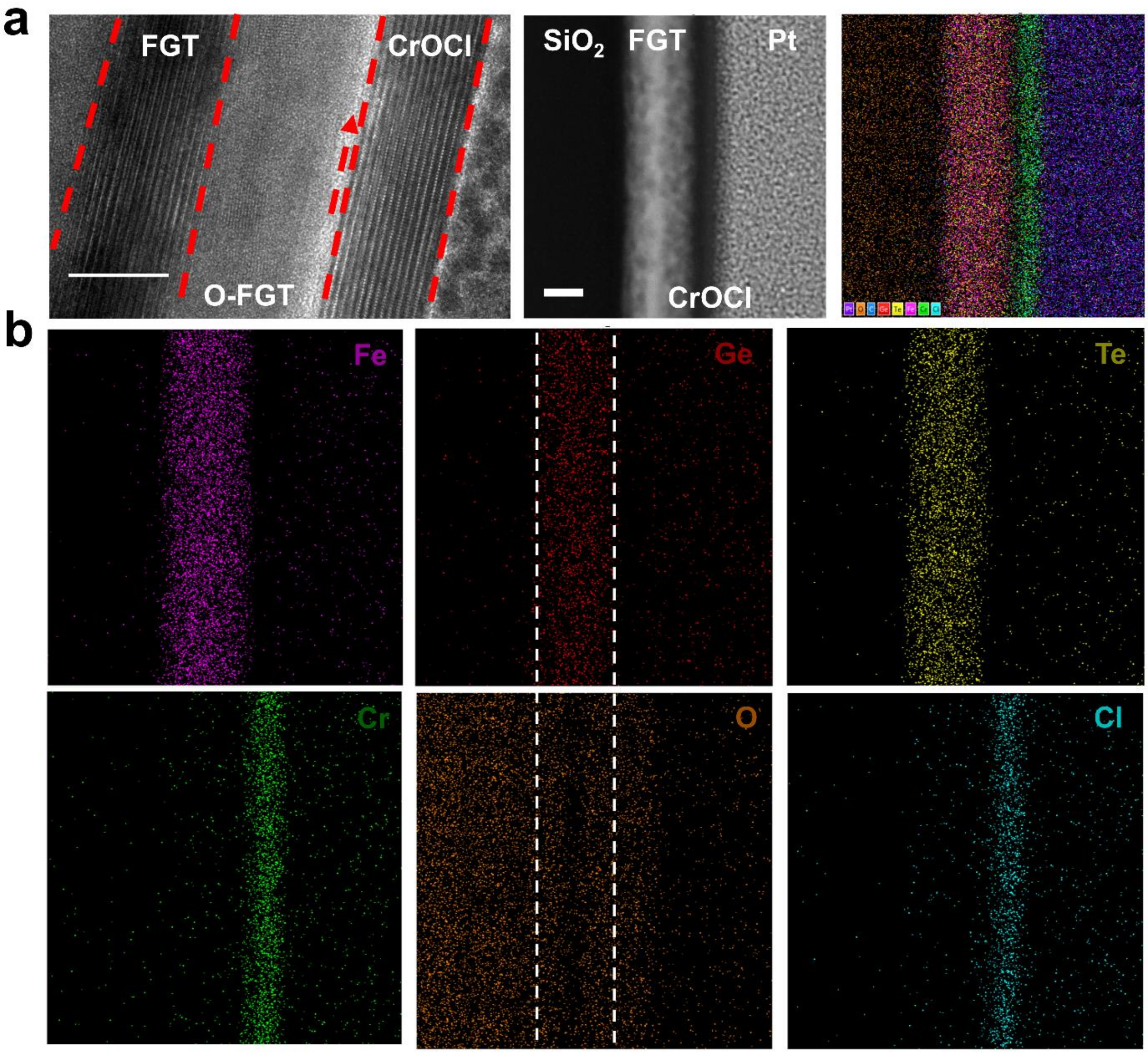


**Figure S8.** a) Cross-sectional HRTEM image (left), STEM image (middle), and a sum of the element distribution (right) of FGT/CrOCl heterostructure exposed in air for 1 week. Scale bar is 10 nm. b) Corresponding element mapping of FGT/CrOCl heterostructure exposed in air for 1 week. The O distribution in FGT is denoted by the white dashed line.

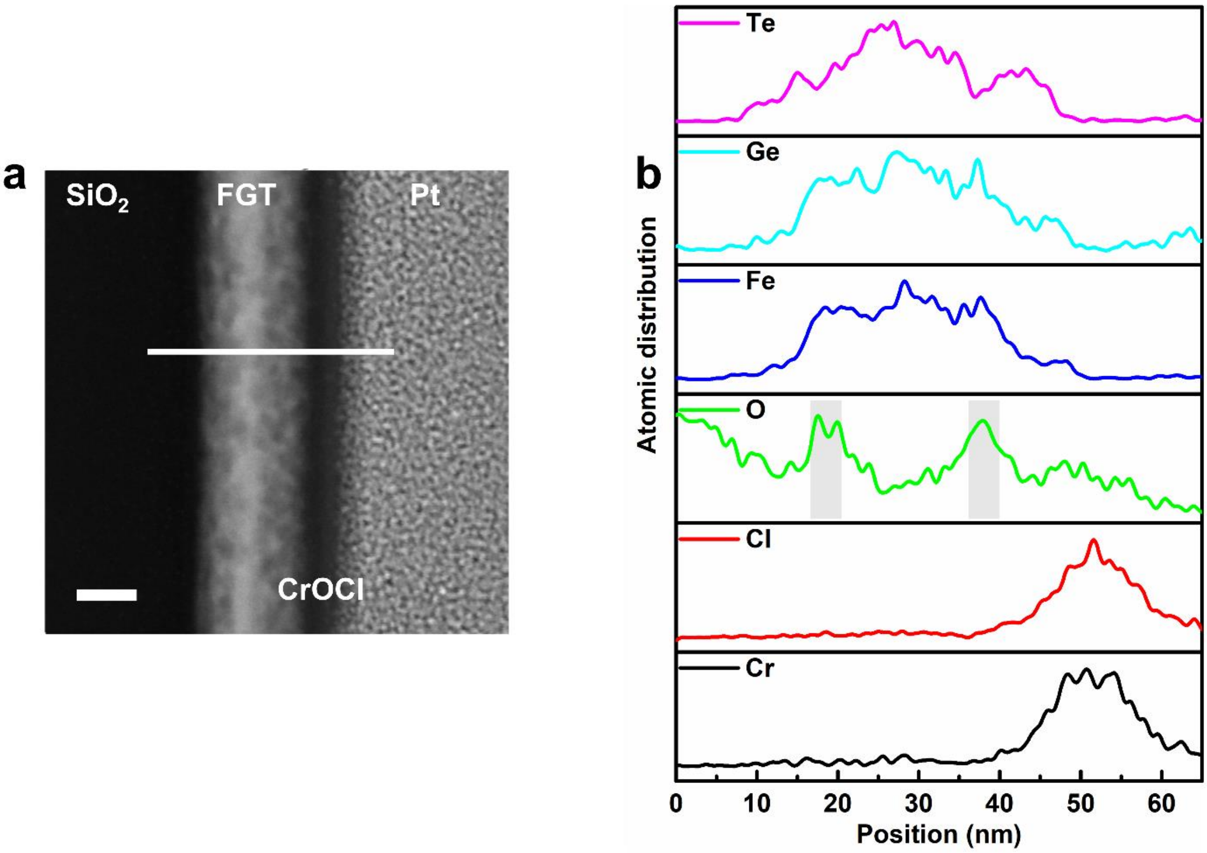


**Figure S9.** a) Cross-sectional STEM image of FGT/CrOCl heterostructure exposed in air for 1 week. b) Element distribution along the white line in Figure S9a. The O distribution near the FGT/CrOCl interface is denoted by the gray bar. Scale bar is 10 nm.

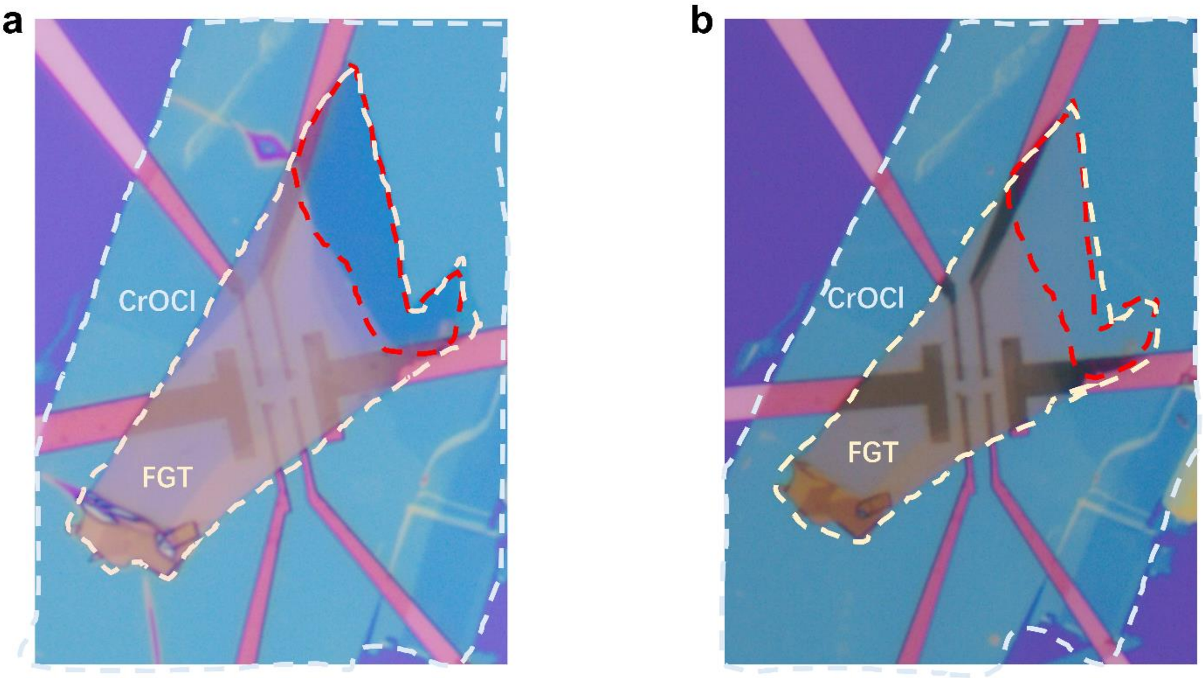


**Figure S10.** OM images of fresh (a) and air-exposed (b) FGT/CrOCl heterostructure. Thin region of FGT is denoted by red dashed line.

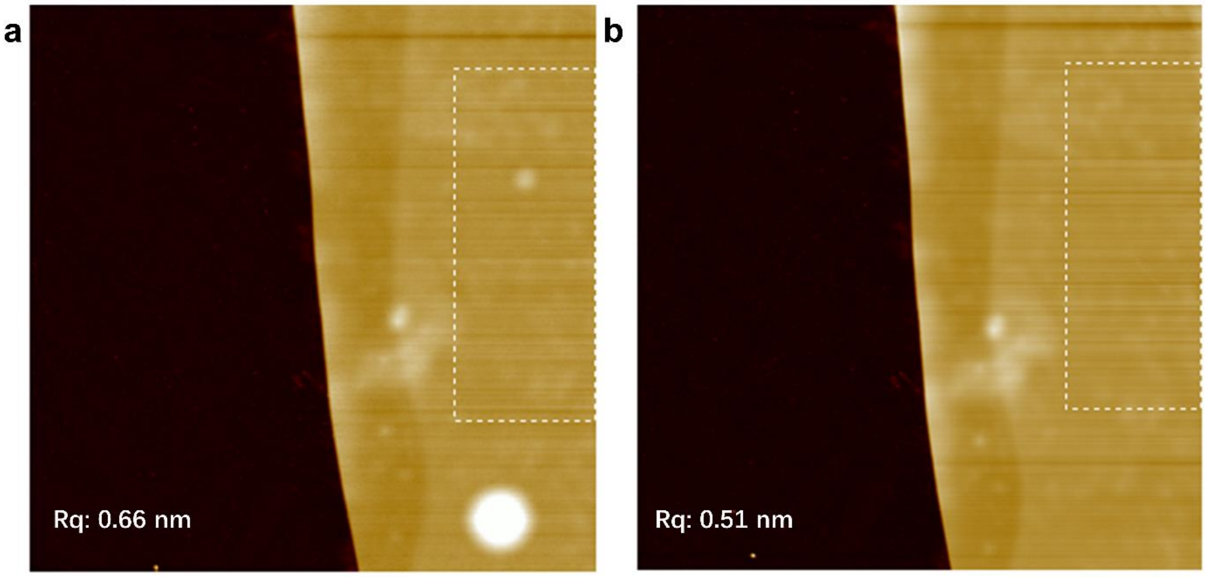


**Figure S11.** The atomic force microscope images of the oxidized (a) and fresh (b) FGT flakes. The calculated root mean square of the roughness of the region marked by white dashed rectangle is listed in the picture.

**Table S1.** The estimation of $J_{AFM\text{-}FM}$ of the samples used in this work.

| $t_{FM}$ [nm] | $t_{AFM}$ [nm] | cooling field [T][a)] | air exposure[b)] | $J_{AFM\text{-}FM}$ [mJ m$^{-2}$] |
|---|---|---|---|---|
| 27 | 28 | 1 | N | 0.14 |
| 27 | 28 | 1 | Y | 0.46 |
| 27 | 10 | 1 | N | 0.22 |
| 17 | 10 | 1 | N | 0.18 |
| 40 | 10 | 1 | N | 0.17 |
| 35 | 12 | 1 | Y | 0.65 |
| 35 | 12 | 0.5 | Y | 0.59 |
| 35 | 12 | 0.1 | Y | 0.33 |

a) The bias field used here is the average value of bias fields measured at 3 K under PFC and NFC of the same magnitude of cooling field.

b) "Y" represents that the sample is exposed to air for a period of time, while "N" represents not.

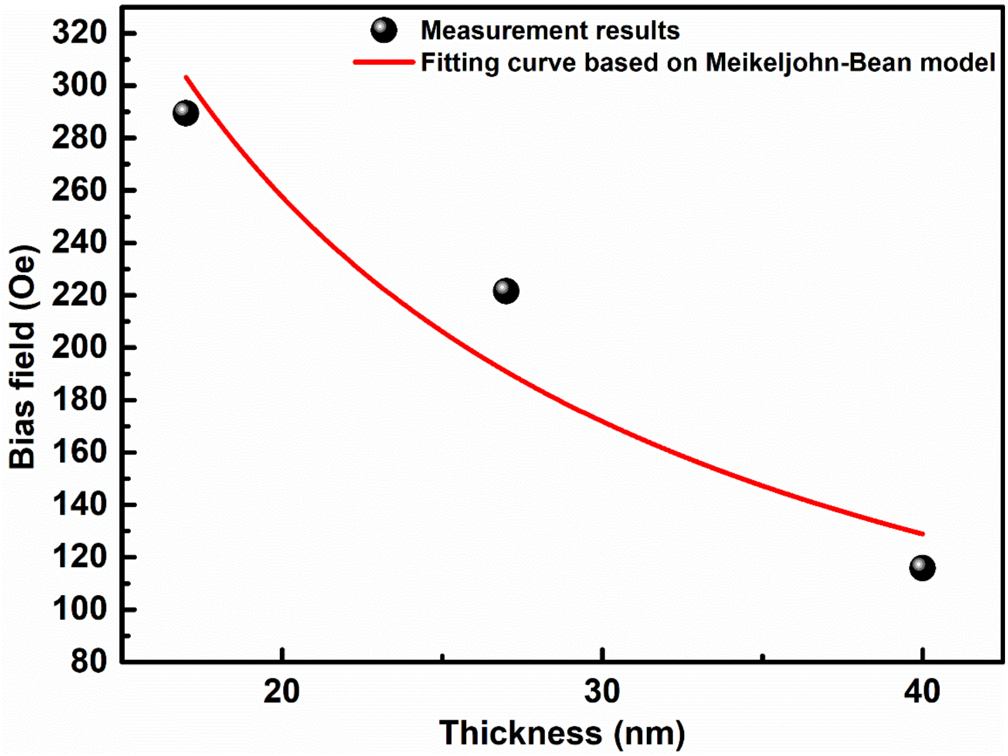


**Figure S12.** The bias field at 3 K as a function of the FGT thickness. The dots are the experiment results, and the red line is the fitting result based on Meiklejohn-Bean model.

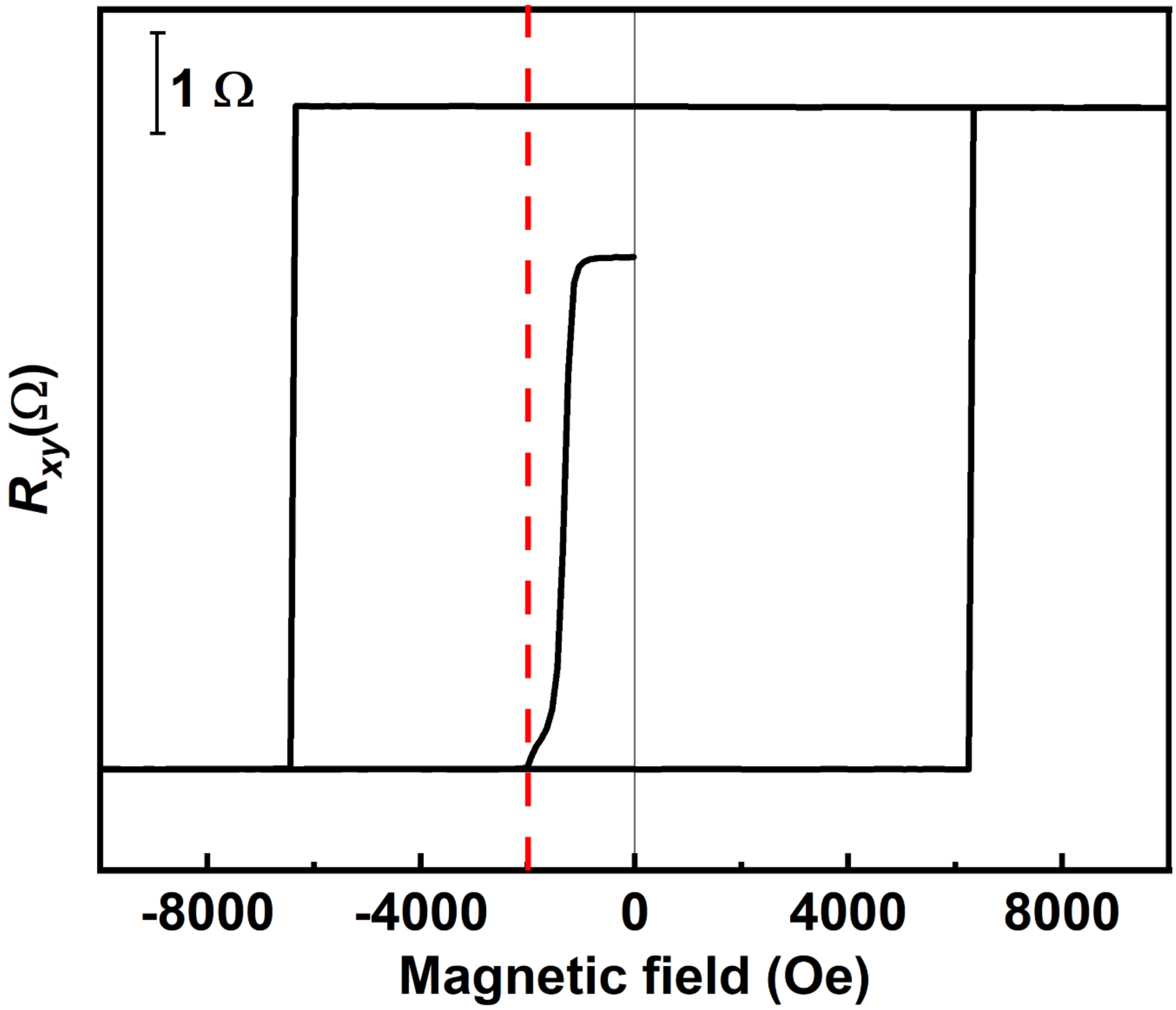


**Figure S13.** Hysteresis loop with initial magnetization curve of FGT/CrOCl heterostructure measured at 3 K under ZFC. The saturation field in initial magnetization curve is denoted by the red dashed line, which is around -2000 Oe.

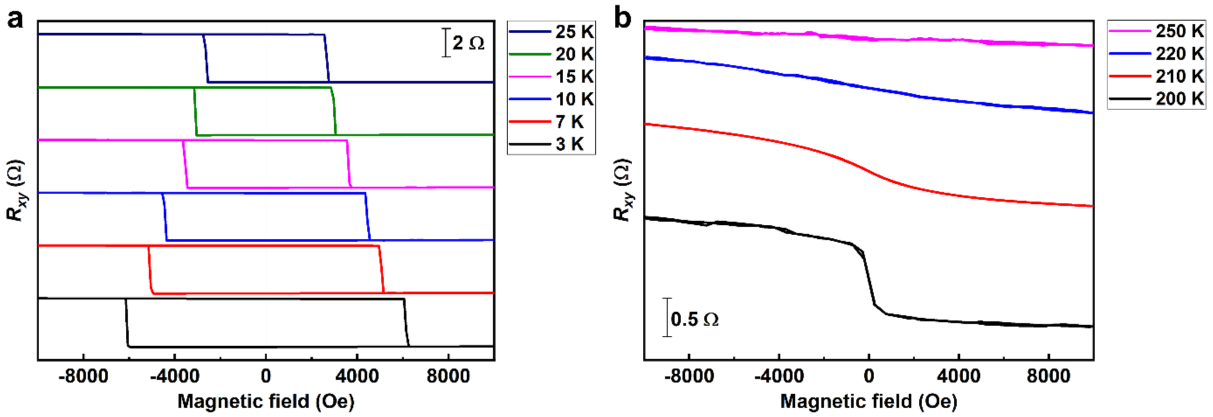


**Figure S14.** Hysteresis loops of pure FGT measured at low temperatures (a) and high temperatures (b).

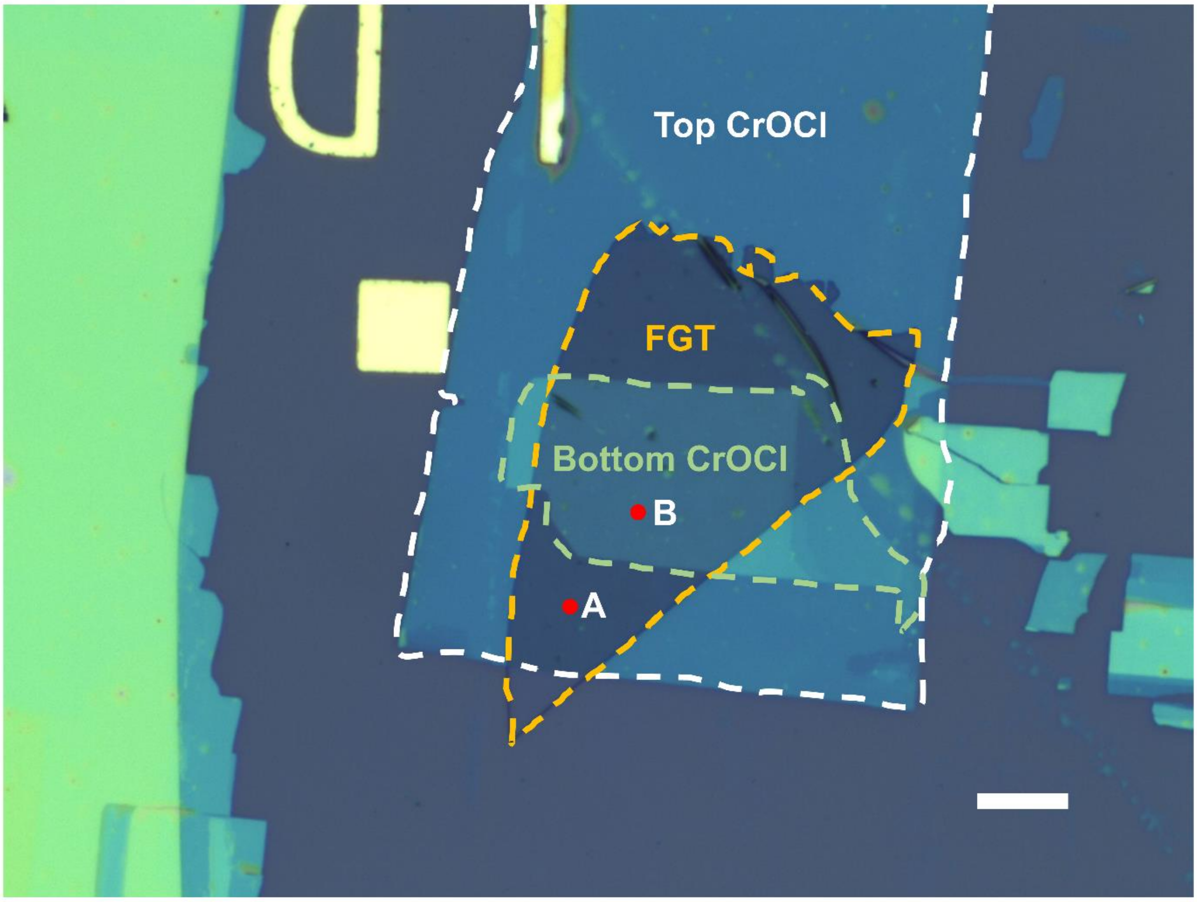


**Figure S15.** OM image of sample used in the RMCD measurements. Scale bar is 10 μm.

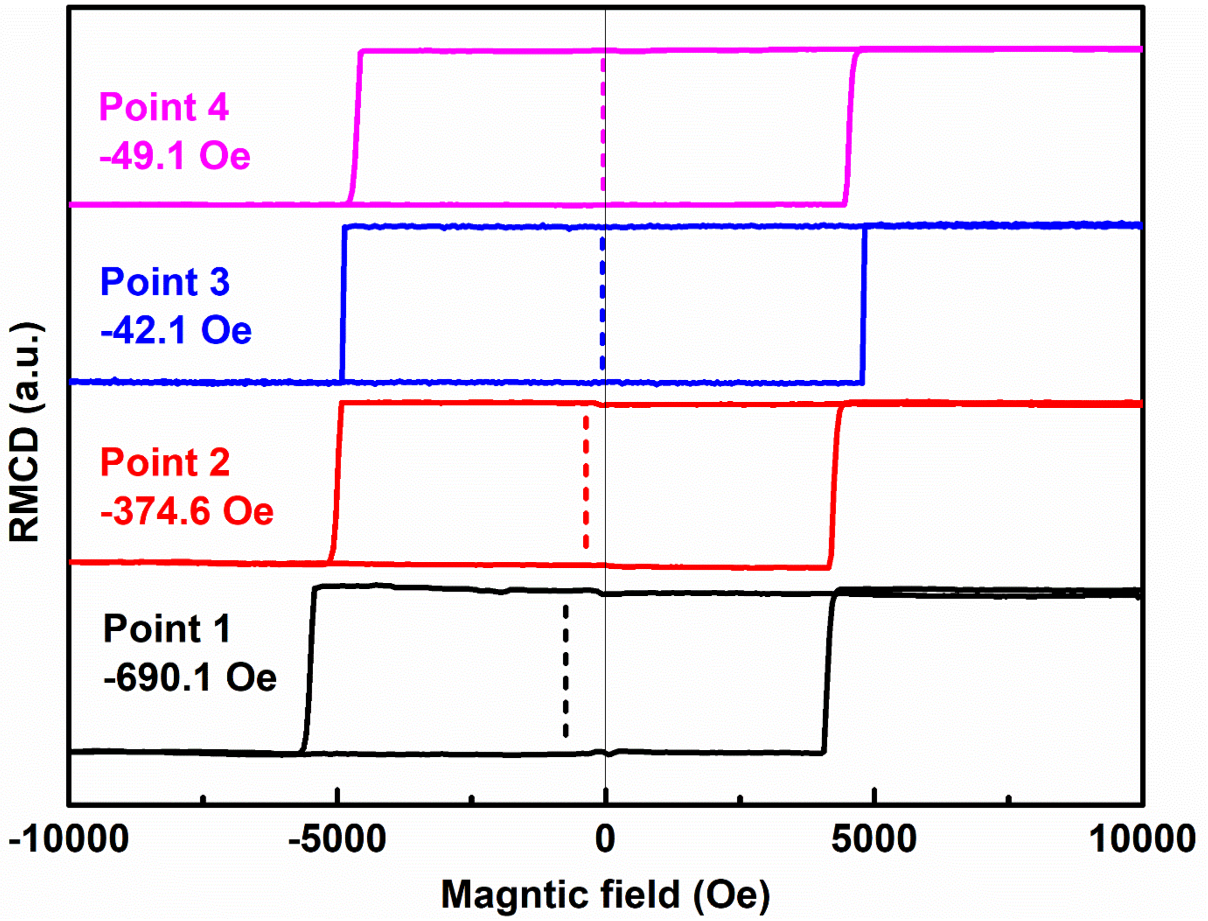


**Figure S16.** The hysteresis loops measured at 3 K in different regions of the FGT/CrOCl heterostructure using RMCD measurements. The cooling field is 1 T for all these measurements.

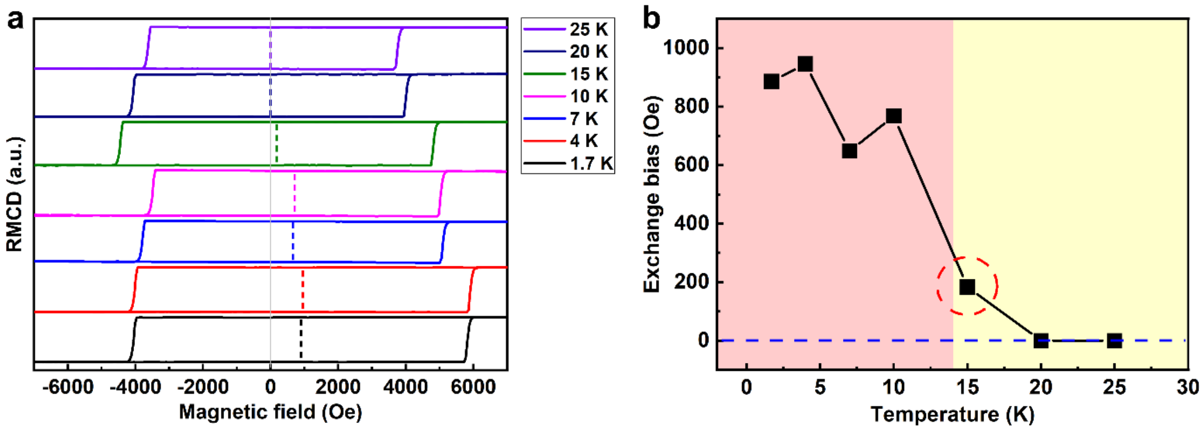


**Figure S17.** a) Field-dependent RMCD signals of CrOCl/FGT/CrOCl heterostructure (point B in Figure S14) measured at different temperatures under the cooling field of -1 T. b) Temperature dependence of bias field. The bias field at 15 K is marked by the red dashed circle.

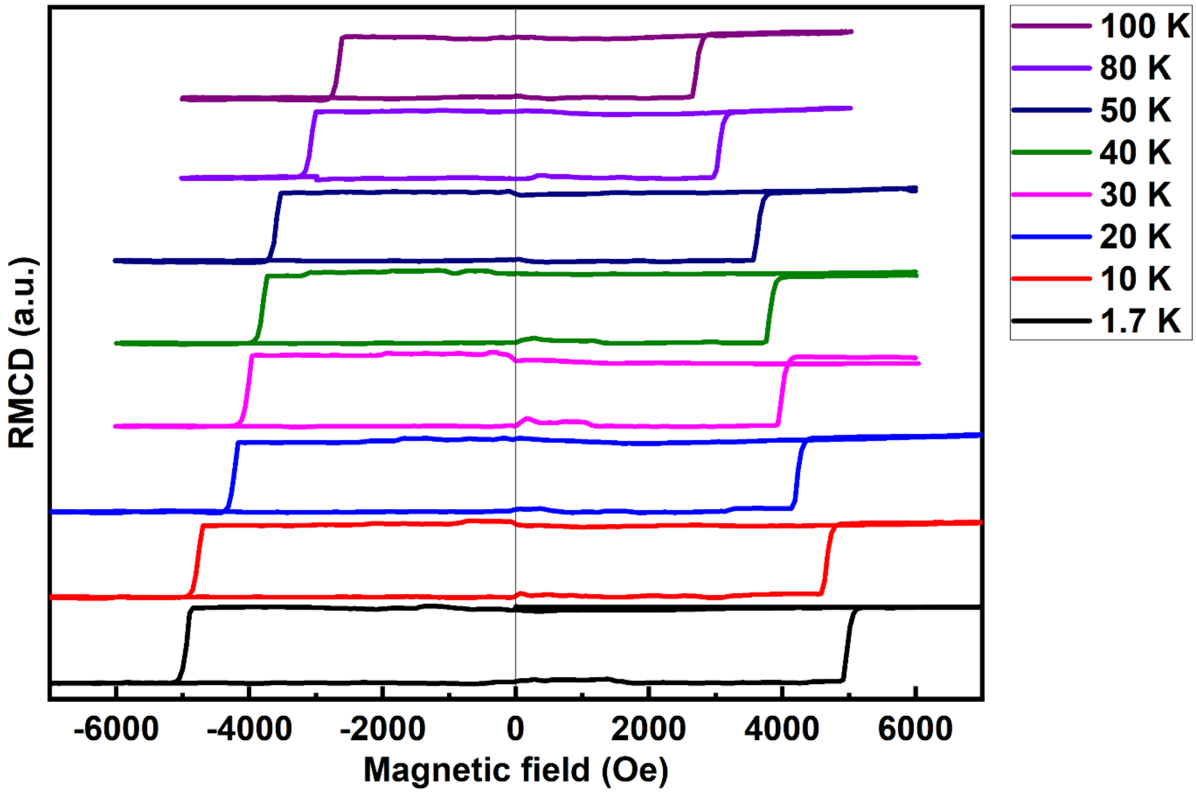


**Figure S18.** Field-dependent RMCD signals of pure FGT measured at different temperatures (1.7 ~ 100 K).
